# Supplementary material for: Asynchronous magnetic resonance elastography: Shear wave speed reconstruction using noise correlation of incoherent waves
Source: Magn Reson Med. 2022 Oct 27;89(3):990–1001. doi: 10.1002/mrm.29502 (PMC9792433; doi:10.1002/mrm.29502)
Supplement: Supplementary file 1 — DATA S1 MATLAB code used to generate the numerical simulations of Figure 1 [file MRM-89-990-s002.zip › k-Wave/helpfiles/extractAmpPhase.html]

extractAmpPhase :: Functions (k-Wave)


# extractAmpPhase

Extract amplitude and phase from CW signals.

## Syntax

```
amp = extractAmpPhase(data, Fs, source_freq)
amp = extractAmpPhase(data, Fs, source_freq, ...)
[amp, phase] = extractAmpPhase(data, Fs, source_freq)
[amp, phase] = extractAmpPhase(data, Fs, source_freq, ...)
[amp, phase, freq] = extractAmpPhase(data, Fs, source_freq)
[amp, phase, freq] = extractAmpPhase(data, Fs, source_freq, ...)
```

## Description

`extractAmpPhse` extracts the amplitude and phase information at a specified frequency from a vector or matrix of time series data. By default the time dimension is set to the highest non-singleton dimension. The amplitude and phase are extracted from the frequency spectrum, which is calculated using a windowed and zero padded FFT. The values are extracted at the frequency closest to `source_freq`.

## Inputs

|  |  |
| --- | --- |
| `data` | matrix of time signals [s] |
| `Fs` | sampling frequency [Hz] |
| `source_freq` | frequency at which the amplitude and phase should be extracted [Hz] |

## Optional Inputs

Optional 'string', value pairs that may be used to modify the default computational settings.

| Input | Valid Settings | Default | Description |
| --- | --- | --- | --- |
| `'Dim'` | *(integer scalar)* | *first non-singleton dimension* | Dimension over which the signals vary in time. |
| `'FFTPadding'` | *(scalar)* | `3` | Scaling parameter used to zero pad the FFT, where the FFT length = FFTPadding \* Nt. |
| `'Window'` | *(string)* | `'Rectangular'` | Parameter string controlling the window type used to filter the signal before the FFT is taken. Any valid input types for `getWin` may be used. |

## Outputs

|  |  |
| --- | --- |
| `amp` | amplitude [au] |
| `phase` | phase between 0 and 2\*pi [rad] |
| `freq` | closest frequency to `source_freq` at which amplitude and phase are extracted [Hz] |

## See Also

`createCWSignals`, `toneBurst`
